# Prevalence of* Plasmodium falciparum* Malaria among Pregnant Students in Dodoma Region, Tanzania: No Cases Have Been Detected

**DOI:** 10.1155/2015/473203

**Published:** 2015-11-19

**Authors:** Karen N. Zablon, Charles Kakilla, Tatiana Lykina, Victoria Minakova, Alphaxad Chibago, Zanda Bochkaeva

**Affiliations:** ^1^University of Dodoma, P.O. Box 259, Dodoma, Tanzania; ^2^University of Dodoma Health Centre, P.O. Box 259, Dodoma, Tanzania

## Abstract

Malaria in pregnancy, being often asymptomatic, is a major problem in endemic African countries. It is characterized by anemia and placental malaria leading to poor pregnancy outcomes. In 2001 Tanzania adopted an intermittent-preventive treatment of malaria in pregnancy (IPTp) policy, which recommends receiving doses of antimalarial drugs every planned visit to the antenatal care centre (ANC), starting from the second trimester. Currently the policy is valid across the whole country, regardless that there are regions with very low malaria endemicity in Tanzania, such as Dodoma region. The current study aimed to show the real prevalence of malaria among young pregnant women in Dodoma region, by measuring the prevalence of malaria among University of Dodoma (UDOM) students, and to describe the social health care features of student female community. Two methods of malaria diagnostic, microscopy, and rapid test, as well as retrospective inspection of ANC registry book, showed the very low prevalence of malaria disease among pregnant students, approximately 0.3%. Additionally, the sociodemographic data from the questionnaires showed that all students use different malaria preventive measures, and most of them have the regular sexual partner. This fact approves the correlation between illiteracy of woman and the risk of malaria infection transmission.

## 1. Background

Malaria is a major worldwide health problem. The disease transmission is ongoing in 97 countries; generally Sub-Saharan Africa is the area most burdened by malaria. In Eastern and Southern Africa, there are 179 million people in the 12 countries at high risk [1]. Between 2006 and 2012, the reported rate of malaria-related deaths in Tanzania decreased by more than a half; nonetheless, there were still some 1,5 million cases of malaria reported in 2013 [2].

In Tanzania, more than 80% malaria cases are linked to infection with* Plasmodium falciparum* [3]. The species is dangerous for pregnant woman as it typically invades large numbers of erythrocytes, causing severe anemia, which is crucial in pregnancy. Moreover, the specific antigen, located on the* P. falciparum*-infected erythrocyte's surface (PfVAR2CSA), assists in the accumulation of parasites in the placenta [4–12]. This condition disrupts the normal exchanges between mother and the fetus, resulting in poor pregnancy outcomes [13]. In high transmission settings, parasites are present in the placenta and contribute to maternal anemia which may lead to low birth weight and even fetus death. In low transmission settings, malaria in pregnancy is associated with anemia and increased risk of severe malaria, spontaneous abortion, stillbirth, prematurity, and low birth weight of the newborn child.

In endemic areas, malaria in pregnancy is often asymptomatic, especially in cases of primigravidae; additionally the parasite may be undetectable in peripheral blood smears, causing the delay in disease treatment [14]. In 2001, aiming to decrease the disease cases in pregnancy, Tanzania adopted the IPTp policy, which recommends sulfadoxine-pyrimethamine (SP) for all pregnant women, starting from the second trimester, at each scheduled ANC visit until the time of delivery. Later in 2006 the observed decline in parasite sensitivity to SP resulted in the policy being updated [15–18] and the SP was recommended to be replaced with artemisinin-based combination therapy (ACT). The major limiting factor of using ACT immediately was the cost [19, 20]. As pregnant women are systematically excluded from clinical trials, the safety of any antimalarials in pregnancy, as well as teratogenic effect, is unclear [21].

Most of research investigations of malaria disease and* Plasmodium* prevalence in Tanzania were conducted in areas of high transmission, specifically the Lake Victoria region, the south of the country, and coastal regions [22–24]. The malaria situation in central part of Tanzanian mainland has been barely studied. Nonetheless the IPTp policy continues in all areas of Tanzania, regardless of rates of transmission and diagnostic results in pregnant women. Every woman, visiting the ANC during pregnancy, continues to receive obligatory antimalarial treatment.

Dodoma region is in the central part of Tanzania. The area features a semiarid climate with relatively warm temperatures throughout the year. While average highs are consistent throughout the year, average lows dip to 13°C in July. The average annual precipitation in Dodoma is 570 mm, the bulk of which occurs during its wet season between November and April. The remainder of the year is referred to as the city's dry season. In Sub-Saharan regions the prevalence of malaria infection has been established to be correlated with the humidity of the climate as mosquitos spread in humid weather.

Generally the prevalence of malaria is less than 1.2% in the UDOM locality, with peaks of up to 3% in October and April, linked to students arrival from all over the country after holiday leave (UDOM health centre malaria report, 2014). This fact, as well as infection correlation with the humidity, allows us to consider students at the end of wet season as the most risky group for malaria.

This study aimed to investigate the real prevalence of malaria among pregnant students of UDOM and describe the social health care features of student female community and hence to account for the observed prevalence. The student community may be considered as representative of the progressive, educated new generation of the country and characterize the likely trajectory of social healthcare attitudes of the general population in the future. The results can be considered as a valid proxy for reconsidering the IPTp policy implementation in low risk regions.

## 2. Materials and Methods

### 2.1. Study Sites

The study was conducted in the University of Dodoma Health Centre (UHC). UDOM is the largest university in Tanzania, in 2014/2015 academic year; 19,671 students studied in UDOM. All students live on the campus during the study period, leaving the campus for holidays only. The health centre is located at the area of the university campus, some 8 km east of Dodoma town. The centre provides health care services for all UDOM students, staff members, and people, living in the villages nearby.

Data and blood samples were collected from 1st to 30th April, 2015, the end of wet season when the rate of malaria is at its peak [25].

All 52 women, who visited the ANC during the study period, were recruited, 2 of them were excluded from the study as they were not students. A structured questionnaire with multiple choice, closed questions was used to obtain sociodemographic characteristics and the use of antimalarial preventive measures. The blood samples were used for malaria diagnostic.

### 2.2. Exclusion Criteria

Pregnant women who were not students were excluded from the study. Women with sickle cell anemia or HIV positive would have been excluded but none was present in the recruited group.

### 2.3. Determination of Sample Size

The sample size was calculated using the following formula: *N* = *Z*_1−*α*/2_^2^ *p*(1 − *p*)/*d* ^2^ with a prevalence of malaria of 3% (data from UHC malaria report) and a precision rate of 5%, allowing an attrition rate of 5% [26]. The minimum sample size for this study was calculated to be 45, and therefore 50 women were recruited. These were all women, who visited ANC over a study period from April 1 to April 30, 2015 (except excluded individuals).

### 2.4. Questionnaires Data

The questionnaire with multiple choice, closed questions was developed for pregnant women to obtain information about age, marital status, general health, and methods of antimalarial prevention used. The questionnaire was designed in English and then translated into Kiswahili, the native language of Tanzanians, to avoid misunderstanding of medical terminology. The received data were analyzed to give the sociodemographic characteristic of the pregnant students group.

### 2.5. Blood Sample Collection

Blood samples were collected from a finger prick for laboratory analysis, which included thick blood smears for microscopy, RDT performance, and determination of hemoglobin concentration.

### 2.6. Laboratory Analysis

Microscopy and RDT were used to detect* P. falciparum* infection. Giemsa-stained thick blood smears were used for microscopy. Blood slides were examined using light microscopy at 1,00*∗*magnification. 100 microscopic fields were examined in the thick smear before concluding that blood slide was negative.

Rapid diagnostic tests were also performed. In this study, the SD Bioline Malaria Antigen P.f/Pan detecting HRP2 was used (Lot number 090385).

Determination of the concentration of hemoglobin count was performed with a portable HemoCue device. Anemia was defined by the hemoglobin concentration less than 11 g/dL: Hb < 7 g/dL: severe anemia, Hb 7–9.9 g/dL: moderate anemia, and Hb 10–10.9 g/dL: mild anemia.

### 2.7. Statistical Analysis

Statistical analyses of malaria and anemia prevalence, as well as sociodemographic characteristics, were carried out using the SPSS statistical package.

### 2.8. Trend Analysis

Data varying with time from 2012 to 2014 was collected from the medical offices in order to show the rate of malaria prevalence at the University of Dodoma Health Centre.

### 2.9. Ethical Issues

The study was approved by the University of Dodoma, College of Natural and Mathematical Sciences, in association with UDOM Health Centre. An oral informed consent was obtained from all pregnant students, prior to their involvement in the study and before answering the provided questionnaires.

## 3. Results

### 3.1. Prevalence of Malaria among Pregnant Students of UDOM

The number of pregnant students visiting the ANC during study period included 14% in the first trimester, 52% in the second trimester, and 34% in the third trimester. Women in the second or third trimester of pregnancy had previously visited the ANC at least 6 weeks before the study period. During that visit, some of them had received a dose of SP to prevent malaria. SP is characterized by a relatively long elimination period of 100 hours, pyrimethamine, and 200 hours, sulfadoxine [27].

Both techniques, microscopy and RDT, used in investigation, showed that none of the 50 pregnant women had malarial infection.

The overall malaria prevalence during the period of 3 years, 2012–2014, was 0.3% (Table 1).

### 3.2. Prevalence of Anemia

Of the 50 samples, the average hemoglobin count was 11.51 g/dL, 62% were not anemic, 32% were mildly anemic, and 6% were moderate anemic. No severe cases were observed.

In the previous year (2014), the mean hemoglobin concentration was 11.32 g/dL and the prevalence of anemia was 37%. Of these anemic pregnant women, 42 (18.5%), 39 (17.18%), and 3 (1.32%) had, respectively, mild, moderate, and severe anemia.

### 3.3. Sociodemographic Characteristics

In total, 50 pregnant women from 21 to 40 years old participated in the study: 52% were 21–25 years old, 32% were 26–30, 10% were 31–35, and 6% were 36–40. All 50 subjects were married or had a regular partner, and 70% were primigravidae.

All recruited women were married or had regular sexual partner.

### 3.4. Possession and Use of Insecticide Treated Mosquito Nets and Other Preventive Measures

**Figure 1:**
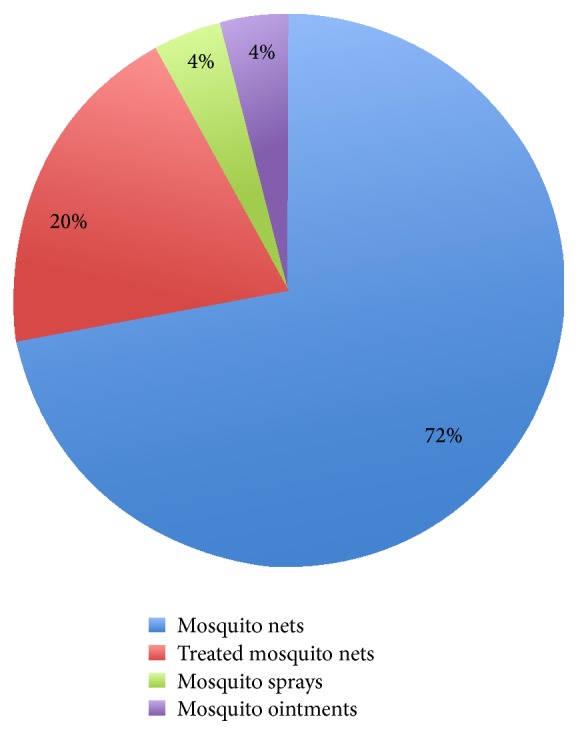
Statistics of malaria preventive measures used by pregnant women, living in UDOM campus.

Of all 50 participants, 92% possessed mosquito nets. Of these 72% used untreated mosquito nets, while 20% had treated mosquito nets. The remaining 8% used other malaria preventive measures: 4% used mosquito sprays and the last 4% used mosquito ointment (Figure 1).

## 4. Discussion

Both methods, RDT and microscopy, used in this study, showed the low prevalence of malaria disease among pregnant students of UDOM. Usually in UHC only microscopy is used for detection of* Plasmodium falciparum* infection. The RDT was additionally applied in this study as some investigations emphasized that the method, based on malaria antigen detection, was more sensitive than microscopy [28]. Comparative studies of* P. falciparum* infection diagnostic methods established that species specific PCR is the most sensitive method for asymptomatic malaria diagnosis in pregnancy [9, 29, 30]. However, currently the PCR method is not available in Dodoma region.

Prevalence of* P. falciparum* infection had been expected to be highest in April because of climate specificity and students arrival from vacations in the middle of the March. Dodoma's dry season ranges from May to November suggesting that this period would reflect mild or low malaria transmission. Selection of April was expected to show the highest rate of malaria infection. According to WHO media centre [31], malaria prevalence increases with the end of rainy season. Additionally, malaria prevalence in pregnancy is said to be associated with age [32–34]. This implied that malaria is more severe in the younger pregnant woman and, aligned with other studies which have shown that, in comparison to older women, younger women may be more susceptible to malaria because they are still in the process of acquiring natural immunity to pregnancy related malaria [14]. Young pregnant women, arriving from different parts of Tanzania, examined in the end of rainy season, were expected to be in the highest risk group for malaria infection yet; even this high risk group, in the most endemic period, showed no cases of malaria.

Zero observation of positive results by both diagnostic techniques confirms that malaria can be easily prevented. Pregnant students showed the proper adherence to malaria preventive measures; they were well aware of the malaria as a disease and its preventive tools. More than 90% of recruited women used mosquito bed nets, which is the cheapest preventive measure in Tanzania, where nets are distributed free to all pregnant women. Use of nets has been established to be the most significant factor in the reduction of malaria infection [35], and these results are a further illustration of the effectiveness of nets. Nevertheless, additional preventive tools, like sprays and ointments, are highly recommended, but they are too expensive for many Tanzanians.

The positive sociodemographic data is an additional factor impacting on the efficient management of individual risk among pregnant students of UDOM. All pregnant students, visiting the ANC during the study, were married or had a regular sexual partner, although it was not a criterion for inclusion in the study. This fact additionally characterizes students as a literate community and supports the correlation between malaria disease and literacy.

Prevalence of anemia for pregnant women at the University of Dodoma for the last year was 37% (UHC, 2014), which is relatively low for an African malaria endemic country. During the study, the prevalence of anemia and malaria was both low. Low prevalence of anemia was observed to be strongly correlated with asymptomatic* P. falciparum* infection [36]. A similar observation was also reported by WHO [37] that* P. falciparum* infection leads to chronic anemia and placental malaria infection, reducing the birth weight and increasing the risk of neonatal death.

Currently pregnant Tanzanians continue to receive obligatory SP, according to the IPTp policy in all regions of the country. The toxicity and safety of the SP treatment regime are still debatable, as it can cause severe cutaneous adverse reactions, teratogenicity, and changes in bilirubin metabolism [27, 38]. The observed low prevalence of malaria and low prevalence of anemia impugns the necessity of receiving SP, as a malaria preventive drug, during pregnancy in Dodoma region.

Malaria is preventable and treatable, and it continues to be associated with illiteracy and poverty, so the received results (from this group of high literacy and relatively low poverty individuals) could have been predicted to differ from the same study in rural regions of the country. This study, only, cannot be considered to draw a conclusion about the whole malaria situation in Tanzania. However, because of the shown low incidence of malaria in pregnancy, even in this high risk group, at least the most sensitive diagnostic method is highly recommended to be used before administration of malaria preventive drugs, especially because the effect of all antimalarials on the fetus is unclear.

## 5. Conclusion 

The findings show the very low infection rates of malaria among pregnant women, studying at the University of Dodoma. Additionally the prevalence of anemia is relatively low. Mosquito nets were the major tool used by recruited women to prevent malaria. The absence of disease cases during the study and the discussed risks of receiving SP in pregnancy cast doubt on the appropriateness of the IPTp policy in regions with low malaria infection rates. Further investigations of malaria prevalence amongst pregnant women, living in rural parts of the Dodoma region, are suggested.

## Figures and Tables

**Table 1:** The number of identified malaria infections among pregnant women during 2012–2014. Source: ANC pregnancy registry books.

Years	Total population size	Malaria cases
2012	90	1
2013	235	0
2014	286	1
